# Mechanisms of Resistance to Photodynamic Therapy (PDT) in Vulvar Cancer

**DOI:** 10.3390/ijms23084117

**Published:** 2022-04-08

**Authors:** Beata Joanna Mossakowska, Somayeh Shahmoradi Ghahe, Dominik Cysewski, Anna Fabisiewicz, Barbara Tudek, Janusz Aleksander Siedlecki

**Affiliations:** 1 Department of Molecular and Translational Oncology, Maria Skłodowska-Curie National Research Institute of Oncology, Roentgena 5, 02-781 Warsaw, Poland; anna.fabisiewicz@pib-nio.pl (A.F.); janusz.siedlecki@pib-nio.pl (J.A.S.); 2 Institute of Biochemistry and Biophysics, Polish Academy of Sciences, Pawińskiego 5a, 02-106 Warsaw, Poland; s.shahmoradi@ibb.waw.pl (S.S.G.); dominikcysewski@gmail.com (D.C.); tudek@ibb.waw.pl (B.T.); 3 Faculty of Biology, Institute of Genetics and Biotechnology, University of Warsaw, Pawińskiego 5a, 02-106 Warsaw, Poland

**Keywords:** photodynamic therapy, photosensitizer, protoporphyrin IX, heme metabolism, multidrug resistance, DNA repair, APE1, combined therapy, APE1 inhibitor

## Abstract

Photodynamic therapy (PDT) is a valuable treatment method for vulvar intraepithelial neoplasia (VIN). It allows for the treatment of a multifocal disease with minimal tissue destruction. 5-Aminolevulinic acid (5-ALA) is the most commonly used prodrug, which is converted in the heme pathway to protoporphyrin IX (PpIX), an actual photosensitizer (PS). Unfortunately, not all patients treated with PDT undergo complete remission. The main cause of their failure is resistance to anticancer therapy. In many cancers, resistance to various anticancer treatments is correlated with increased activity of the DNA repair protein apurinic/apyrimidinic endonuclease 1 (APE1). Enhanced activity of drug pumps may also affect the effectiveness of therapy. To investigate whether multidrug resistance mechanisms underlie PDT resistance in VIN, porphyrins were isolated from sensitive and resistant vulvar cancer cells and their culture media. APE1 activity was measured, and survival assay after PDT combined with APE1 inhibitor was performed. Our results revealed that resistant cells accumulated and effluxed less porphyrins than sensitive cells, and in response to PDT, resistant cells increased APE1 activity. Moreover, PDT combined with inhibition of APE1 significantly decreased the survival of PDT-resistant cells. This means that resistance to PDT in vulvar cancer may be the result of alterations in the heme synthesis pathway. Moreover, increased APE1 activity may be essential for the repair of PDT-mediated DNA damage, and inhibition of APE1 activity may increase the efficacy of PDT.

## 1. Introduction

Vulvar intraepithelial neoplasia (VIN) is a pre-cancerous lesion of the vulva that may occur as a multifocal disease. It also occurs in women younger than 35 years [1,2] and, if remained untreated, carries a risk of development of invasive cancer [1]. VIN is found in 50–70% of patients with vulvar squamous cell carcinoma (VSCC) [2].

Photodynamic therapy (PDT) is one of the less invasive methods [1,2,3] leading to tumor cell death [3,4]. PDT is a valuable alternative method to surgical treatment of VIN [1,2]. It allows for the treatment of a multifocal disease with minimal tissue destruction, maintenance of vulvar anatomy, and excellent cosmetic outcomes [2]. 

**Figure 1:**
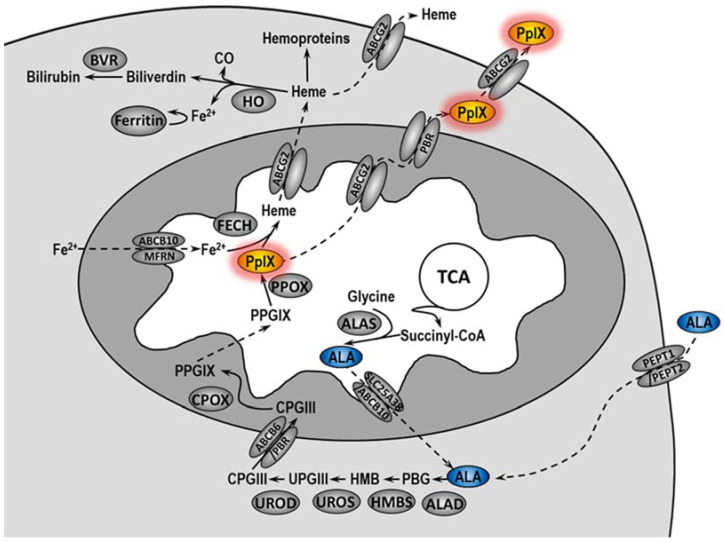
Heme metabolism pathway. ALA is normally synthetized in the mitochondrial matrix by combining glycine with succinyl-CoA in a reaction catalyzed by ALA synthase (ALAS). After passing into the cytoplasm, ALA is converted into coproporphyrinogen III (CPGIII) as a result of numerous changes. Exogenously administered 5-ALA is delivered to the cytoplasm of cells by peptide transporter 1 and 2 (PEPT1 and PEPT2). CPGIII is transported to the intermembrane space of mitochondria where it is converted by coproporphyrinogen oxidase (CPOX) to protoporphyrinogen IX (PPGIX) and then in the mitochondrial matrix to PpIX by protoporphyrinogen oxidase (PPOX). Next, ferrochelatase (FECH) leads to the formation of heme by incorporating iron into PpIX. PpIX and heme can be released from the cell via the ABCG2 transporter. Heme in the cell is used to create hemoproteins or it is degraded by heme oxygenase (HO), leading to the release of biliverdin, CO, and Fe^2+^. Iron ions may be bound and stored by ferritin. Biliverdin is converted to bilirubin by biliverdin reductase (BVR). ABCB10—ABC subfamily B member 10; ALAD—5-aminolevulinic acid synthase dehydratase; HMB—hydroxymethylbilane; MFRN—mitoferrin; PBG—porphobilinogen; PBGD—porphobilinogen deaminase; PBR—peripheral-type benzodiazepine receptor; SLC25A38—solute carrier family member 38; TCA—tricarboxylic acid; UPGIII—uroporphyrinogen III; UROD—uroporphyrinogen decarboxylase; UROS—uroporphyrinogen III synthase.

In PDT, a photosensitizer (PS) applied to the patient accumulates in the tumor, where under the appropriate wavelength of light and oxygen, it makes significant damage, leading to the destruction of cancer cells and tumor vascularity. In addition, PDT induces inflammatory and immune responses against tumor cells [3,4]. Currently, the most commonly used drug in PDT is 5-aminolevulinic acid (5-ALA) [5]. In cells, 5-ALA is converted in the heme biosynthetic pathway to protoporphyrin IX (PpIX), an actual PS (Figure 1) [6,7]. Unfortunately, not all patients treated with PDT undergo complete remission. The literature data indicate that the complete histopathological response rate in VIN patients treated with PDT ranged from 20 to 67%, and the symptom response rate ranged from 52 to 89% [2]. In 2013–2015 at the Maria Skłodowska-Curie National Research Institute of Oncology, VIN patients were treated with PDT using ALA as PS. The efficacy of such treatments was about 90% [8]. 

Resistance to anticancer therapy is the main cause of its failure, leading to tumor progression and poor clinical outcome. The effectiveness of PDT mainly depends on the selective uptake of the photosensitizer by tumor cells, the oxygen level, and the irradiation dose [9]. Multidrug resistance (MDR) may contribute to reduced PS levels in cancer cells. Mechanisms involved in MDR are associated with altered drug uptake, efflux rate, and intracellular distribution. Increased drug pump activity enhances PS efflux from cells and protects them from phototoxicity. Depending on the structure of the photosensitizer, various transporters can remove PS from the cell. Overexpression of ABCG2 transporter leads to increased PpIX efflux from cells and may be associated with the development of resistance to PDT [10].

Accumulation of protoporphyrin IX can be modulated by the activity of heme oxygenase-1 (HO-1). It has been demonstrated that expression of HO-1 increases due to the necessity of heme degradation after incubation of cells with 5-ALA [10]. However, the activity of HO-1 reduces oxidative stress not only through the degradation of heme but also through the activity of its degradation products—biliverdin and bilirubin. Additionally, HO-1 may promote angiogenesis, influence tumor progression, and inhibit anti-cancer immune response [11].

In many cancers, increased activity of the apurinic/apyrimidinic endonuclease 1 (APE1) is correlated with resistance to various anticancer drugs [12,13]. APE1 is a bifunctional enzyme, essential for both DNA repair through the base excision repair (BER) pathway and redox regulation of transcription factors. The major DNA damage after PDT is oxidized base lesion, mainly repaired by BER [14]. Inhibition of the endonuclease repair function of APE1 may be an effective adjuvant in the treatment of cancer [12]. In preclinical studies, inhibition of the DNA repair function of APE1 enhanced the cell death induced by many anticancer drugs, including bleomycin, temozolomide, melphalan, cisplatin, and gemcitabine, as well as IR, and moreover agents such as methyl methane sulfonate (MMS) and H_2_O_2_ [15].

In this study, we analyzed the accumulation of PS within sensitive and resistant vulvar cancer cells and demonstrated that resistance to PDT may result from changes in the heme metabolism pathway. We also show that DNA repair capacity plays an important role in PDT outcome and that inhibition of APE1 increased the PDT effectiveness. 

## 2. Results

### 2.1. Isolation of PDT Resistant Cancer Cells

**Figure 2:**
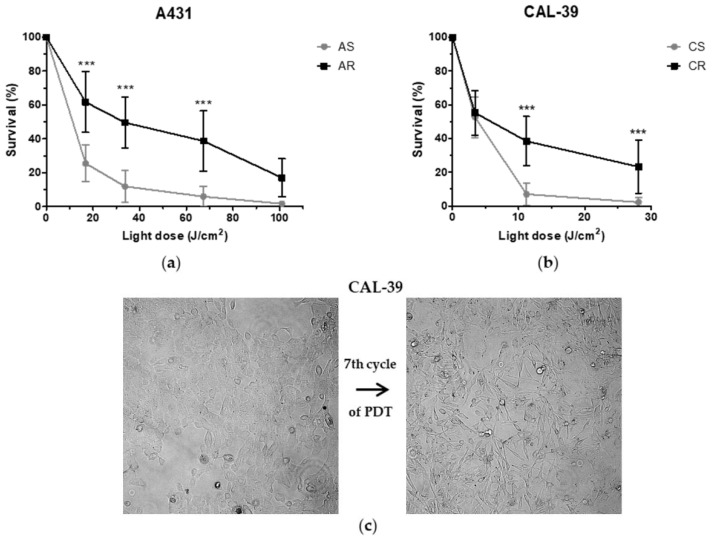
Cell survival in response to PDT. (**a**) Viability of A-431 parental cells that are sensitive to PDT (AS) and isolated resistant cells (AR) after PDT treatment. (**b**) Viability of CAL-39 parental cells, those sensitive to PDT (CS), and isolated resistant cells (CR). Cell survival was expressed as percentage of the non-treated control. Points represent average of at least three independent experiments. Asterisks indicate significant differences between the sensitive and resistant cells calculated by one-way ANOVA followed by the Bonferroni test (*** *p* ≤ 0.001). (**c**) Change in cell morphology after the 7th cycle of PDT in the CAL-39 cell line.

To induce PDT resistance in parental A-431 and CAL-39 vulvar cancer cell lines, cells were subjected a few times to PDT with increasing light dose. Isolation was completed after 10th (light dose of 101 J/cm^2^) and 7th cycles (light dose of 41.5 J/cm^2^) of PDT for A-431 and CAL-39 cells, respectively. AlamarBlue cell viability assay was used to confirm the resistance of isolated cells to PDT. Cells subjected to several cycles of PDT showed significantly higher survival level after PDT than parental cell lines (Figure 2a,b). Moreover, CAL-39 cells after the 7th cycle of PDT changed their morphology. Resistant CAL-39 cells were more elongated (Figure 2c) and detached easier from the plate, whereas resistance to PDT did not induce any morphological changes in A-431 cells (Figure S1c, Supplementary Materials).

### 2.2. Acumulation and Extraction of PpIX

**Figure 3:**
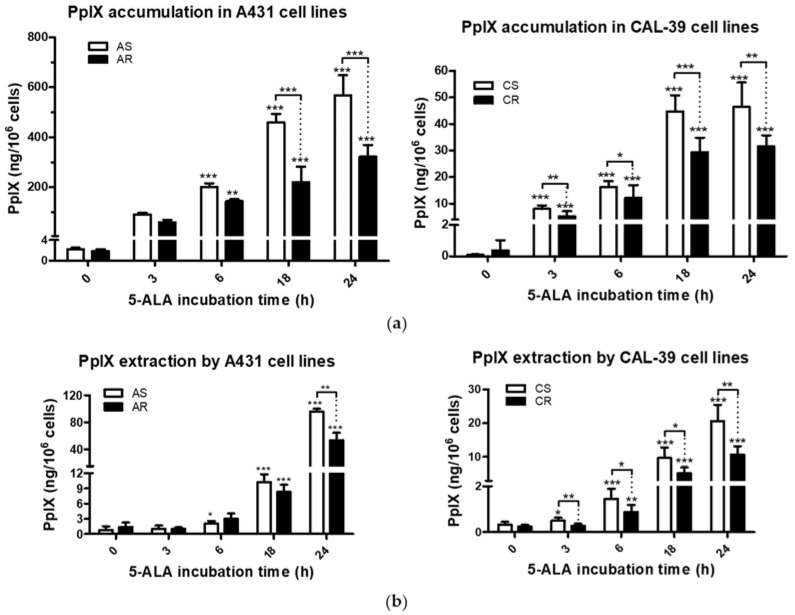
PpIX accumulation and extraction. (**a**) PpIX levels in A-431 and CAL-39 cell lines. (**b**) PpIX levels in culture media collected from above A-431 and CAL-39 cells. The amount of PpIX was calculated from standard curve and presented in ng per 10^6^ cells. Asterisks indicate statistical significance of differences between untreated cells and cells incubated with ALA for various periods of time, or the sensitive and resistant cells calculated by Student’s *t*-test (* *p* ≤ 0.05, ** *p* ≤ 0.01, *** *p* ≤ 0.001).

The efficacy of PDT depends on intracellular accumulation of the photosensitizer by the targeted cells. Increased efflux of PS from cells can protect them from PDT-mediated phototoxicity, leading to the development of resistance. Therefore, the comparison of porphyrin levels in sensitive and resistant cells and in their culture medium was performed. Before 5-ALA treatment, sensitive and resistant cells showed similar levels of PpIX in both the cells and the media in which they were cultured. After administration of 5-ALA, the levels of porphyrins increased in sensitive and resistant cells. However, the intracellular levels of PpIX were lower in PDT resistant cells compared to their parental, sensitive cells (Figure 3a). In culture media of sensitive cells, a significant increase in porphyrin levels was detected after shorter incubation times with 5-ALA compared to that in the resistant cells (6 h and 18 h in AS and AR, respectively, and 3 h and 6 h in CS and CR, respectively; Figure 3b). The number of porphyrins detected in media of sensitive cells was higher than in media of resistant cells (Figure 3b). The reduced levels of porphyrins in PDT-resistant cells and their media suggest an impairment in the synthesis of porphyrins or higher degradation rate of porphyrins rather than their efflux. The reduced levels of PpIX in resistant cells may be at least one of the reasons for lower PDT efficacy.

### 2.3. Assessing the Levels of PPOX, FECH, and HO-1 

Differences in heme biosynthetic pathway between non-malignant and malignant cells lead to accumulation of PpIX preferentially in tumors. Decreased activity of ferrochelatase (FECH), a rate-limiting enzyme that is responsible for insertion of Fe^2+^ into PpIX and limited availability of iron, contributes to increased PpIX accumulation. Additionally, enhanced activity of enzymes leading to the production of PpIX, such as aminolevulinate dehydratase (ALAD), uroporphyrinogen decarboxylase (UROD), and hydroxymethylbilane synthase (HMBS), has been observed in tumor cells [7]. On the other hand, PDT-resistant cells can develop alterations in enzymes involved in heme pathway and biosynthesis of PpIX. These alterations lead to the reduction of protoporphyrin levels and higher proportion of hydrophilic porphyrins. Hydrophilic porphyrins such as coproporphyrin and uroporphyrin are poor photosensitizers [10]. In this study, the amount of protoporphyrinogen oxidase (PPOX), FECH, and HO-1 enzymes were assessed in sensitive and resistant cells, before and after PDT. 

**Figure 4:**
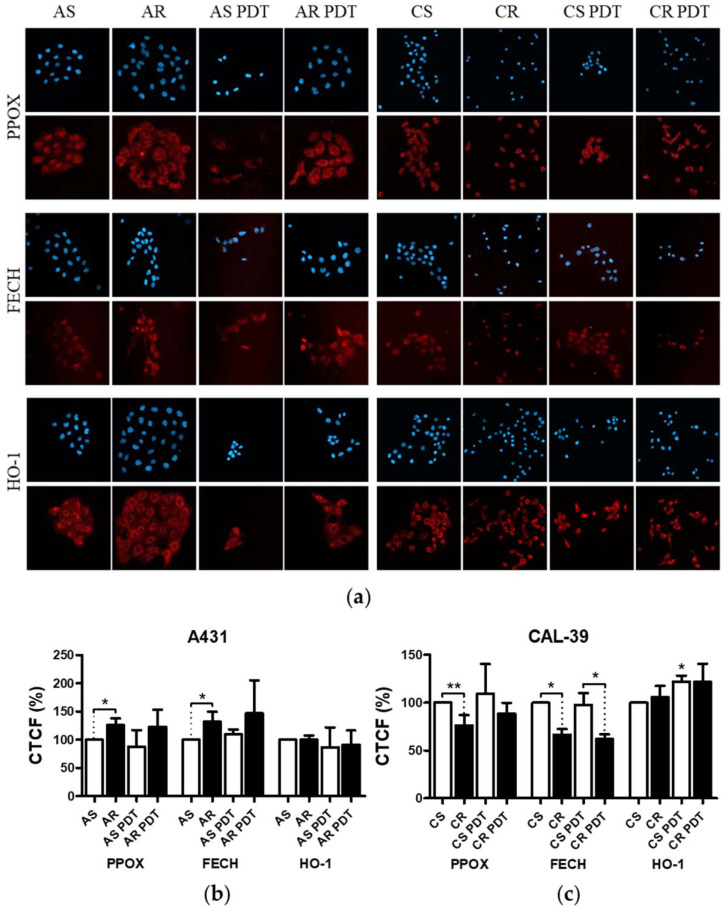
Analysis of the levels of PPOX, FECH, and HO-1. (**a**) Images of stained cells, before and after PDT treatment. (**b**) Graph with percentage values of corrected total cell fluorescence (CTCF) of untreated, parental cells. (**c**) Graph with percentage values of corrected total cell fluorescence (CTCF) of CAL-39 cell lines. Asterisks indicate the statistical significance of differences between cells after PDT and untreated control, and between sensitive and resistant cells calculated by Mann–Whitney test (* *p* ≤ 0.05, ** *p* ≤ 0.01).

Immunofluorescence assay showed an increased amount of PPOX and FECH in AR cells compared to AS, as well as a decreased amount of these enzymes in CR compared to CS. Comparison of HO-1 levels between sensitive and resistant cells did not show any changes. After PDT, no changes in PPOX, FECH, and HO-1 levels were observed in sensitive and resistant A-431 cells and resistant CAL-39 cells. In contrast, the amount of HO-1 increased in CS cells in response to PDT (Figure 4). These results indicate that the decreased levels of photosensitizer in resistant cells is due to changes in PpIX synthesis rate. Resistant CAL-39 cells can produce PS with lower rate, which may lead to weaker response to PDT. In contrast, PDT-resistant A-431 cells can produce PpIX and convert it to heme faster than a sensitive parental line. However, the lack of changes in HO-1 expression may suggest that the rate of heme degradation is not changed.

### 2.4. Protein Profile of PDT-Sensitive and -Resistant Cells

**Figure 5:**
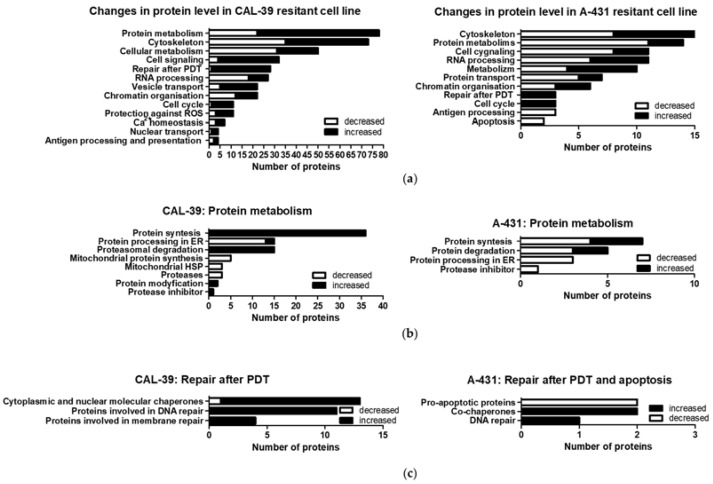
Changes in protein abundance in PDT-resistant cells as compared to the therapy-sensitive, parental cells. (**a**) Proteins whose level was changed in PDT-resistant cells compared to parental, sensitive cell lines, were grouped by processes in which they participate. (**b**) Analysis of proteins involved in protein metabolism. (**c**) Analysis of proteins that may participate in repair after PDT and apoptosis.

For further characterization of PDT-resistant cells, proteomic profiles of parental and resistant cell lines were established by mass spectrometry. The list of identified proteins and their fold change are shown in Tables S1–S5 (Supplementary Materials). Proteins with altered abundance in PDT-resistant cells compared to the sensitive parental cells were grouped by processes in which they participate (Figure 5a). One of the most altered group of proteins was the one that included proteins involved in protein metabolism (Figure 5b). Moreover, this analysis also revealed changes in proteins that may participate in repair after PDT and apoptosis (Figure 5c). 

### 2.5. APE1 Activity Assay 

PDT mainly induces oxidative DNA damage, which, if remaining unrepaired, can lead to the accumulation of dangerous damages, such as double-stranded DNA breaks, as well as DNA–protein and DNA–DNA crosslinks. Repair of oxidative DNA damage takes place through the BER pathway in which the APE1 enzyme plays a crucial role. Increased activity of this protein is associated with a poorer response to treatment. Therefore, the activity of repair function of APE1 was assessed in untreated sensitive and resistant cells and in response to PDT. 

**Figure 6:**
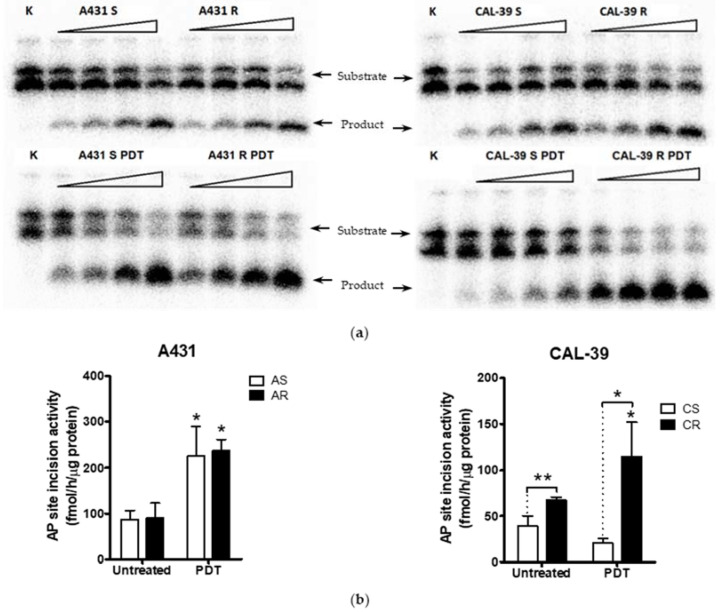
APE1 endonuclease activity assay. (**a**) PhosphorImages of gels with separated products of reaction mediated by protein extracts isolated from the cells before and after PDT. (**b**) Quantification of APE1 endonuclease activity in A-431 and CAL39 cell lines. APE1 activity is expressed as fmol of product per hour per µg of protein extract isolated from the cells. Asterisks indicate statistical significance of differences between untreated cells and cells that were subjected to PDT, and between sensitive and resistant cells, calculated by the Mann–Whitney test (* *p* ≤ 0.05, ** *p* ≤ 0.01).

No difference in APE1 endonuclease activity was observed between sensitive and resistant A-431 cells. However, both AS and AR showed increased activity of this enzyme after PDT (Figure 6a). These results suggest that APE1 DNA repair function is important for diminishing the PDT effects. 

In the contrary, CAL-39 resistant cells showed higher APE1 endonuclease activity than their parental cells. In response to PDT, this activity was further increased in resistant cells (Figure 6b). Therefore, in the CAL-39 cell line, increased APE1 activity may be essential not only for the repair of PDT-mediated DNA damage, but also to develop the resistance. 

### 2.6. Sensitization of PDT-Resistant Cells by Inhibition of APE1 DNA Repair Function

Increased APE1 endonuclease activity seems to be important for reducing the PDT effects in both resistant cell lines. Moreover, increased activity in CAL-39 cell line may be associated with development of resistance to the therapy. Therefore, to verify a hypothesis that inhibition of APE1 DNA repair function may sensitize resistant cancer cells to PDT, the effect of AR03, a specific inhibitor of APE1, alone and in combination with PDT was tested on the PDT-resistant cells.

**Figure 7:**
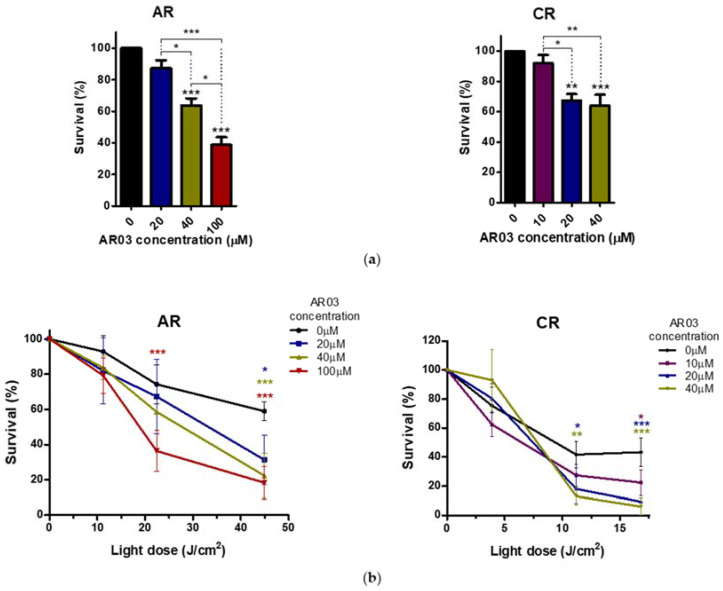
The effect of APE1 inhibition on PDT-resistant cancer cells. (**a**) AR03 effect on cancer cell lines. (**b**) Sensitization of cancer cells resistant to PDT with APE1 inhibitor. Statistical significance was calculated by one-way or two-way ANOVA followed by the Bonferroni test (* *p* ≤ 0.05, ** *p* ≤ 0.01, *** *p* ≤ 0.001).

AR03 decreased the survival of PDT-resistant cells (Figure 7a) and increased the mortality of resistant cells subjected to PDT (Figure 7b). These results indicate that inhibition of APE1 endonuclease activity can effectively sensitize resistant cells to PDT and suggest a positive effect of combining PDT with APE1 inhibitor in treatment of vulvar cancer cells that are resistant to PDT. 

### 2.7. Effect of APE1 Inhibition on Non-Cancer Cell Lines 

**Figure 8:**
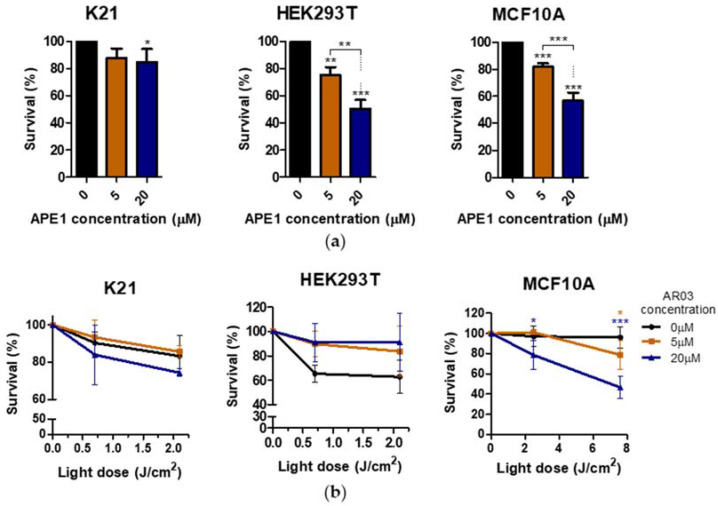
The effect of APE1 inhibition on non-cancer cell lines. (**a**) AR03 effect on K21, HEK293T, and MCF10A cells. (**b**) Influence of APE1 inhibitor combined with PDT treatment on K21, HEK293T, and MCF10A cells. Statistical significance was calculated by one-way or two-way ANOVA followed by Bonferroni test (* *p* ≤ 0.05, ** *p* ≤ 0.01, *** *p* ≤ 0.001).

Due to the positive effect of the APE1 inhibitor on PDT in resistant cancer cell lines, the effect of AR03 alone and in combination with PDT was tested on non-neoplastic cells. The applied doses of APE1 inhibitor and PDT were lower because non-cancer cells accumulate less drugs than cancer cells. Moreover, during the PDT treatment, only tumor and its surrounding tissue are exposed to light. Survival tests were performed on human epithelial MCF10A, embryonic kidney HEK293T, and K21 fibroblastic cells. As shown in Figure 8a, application of AR03 decreased the survival of non-neoplastic cells in all tested lines. Conversely, a combination of AR03 with PDT significantly reduced survival only in the MCF10A line (Figure 8b). These results suggest that the level of toxicity of combined therapy may depend not only on applied doses of APE1 inhibitor and PDT, but also on the type of treated tissue.

### 2.8. Effect of AR03 on PPOX, FECH, and HO-1 Levels

APE1 through its endonuclease activity may participate not only in DNA repair but may also have influence on gene expression profile [16]. Thus, inhibition of APE1 activity may contribute to a better PDT response not only by inhibiting BER. In previous experiment, we showed that changes in heme metabolism (Figure 4) may affect the resistance to PDT. Therefore, the effect of AR03 on previously tested heme metabolism enzymes was examined in PDT-resistant cell lines.

**Figure 9:**
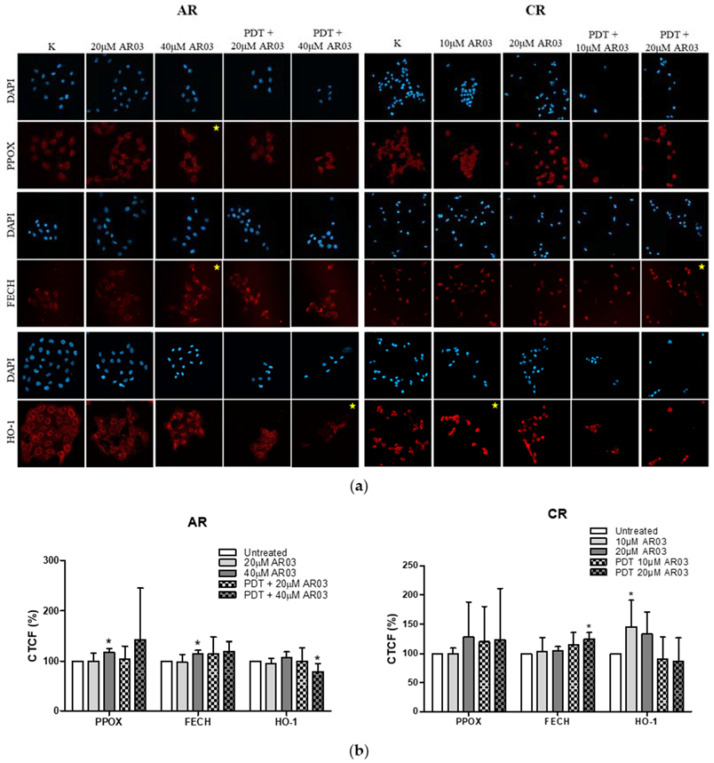
Effect of AR03 on PPOX, FECH, and HO-1 levels. (**a**) Images of stained cells before treatment, after AR03 treatment, or after PDT combined with AR03 treatment. (**b**) Graphs with percentage values CTCF of untreated cells, resistant to PDT. Asterisks indicate the statistical significance of the differences between cells after treatment and untreated control calculated by the Mann–Whitney test (* *p* ≤ 0.05).

Higher concentration of AR03 (40 µM) caused significant increase in PPOX and FECH levels, and its combination with PDT led to a decrease in HO-1 levels in resistant A-431 cells. On the other hand, in resistant CAL-39 cells, the APE1 inhibitor at lower concentration (10 μM) increased the amount of HO-1, and at a higher concentration (20 μM) and in combination with PDT increased the FECH level (Figure 9). These results suggest that inhibition of APE1 endonuclease activity may influence the efficacy of PDT, not only by inhibiting DNA repair but also by influencing heme metabolism.

## 3. Discussion

To achieve a good treatment effect, patients may require more than one PDT cycle [1,17]. On the other hand, the long-term therapy can lead to the development of resistance. Repeated cycles of treatment insufficient to kill all cancer cells lead to PDT failure (Figure 2). The acquired resistance arises as a result of accumulated changes in cells that enable them to survive [10,18,19].

Efficacy of PDT depends, among others, on the ability of tumor cells to accumulate PS within the cells [9,10]. Previous studies have shown that the higher activity of drug pumps reduces intracellular PS level and contributes to PDT resistance [10,18]. However, 5-ALA-treated A-431 and CAL-39 resistant cells accumulate and efflux less photosensitizer, PpIX, than A-431 and CAL-39 sensitive cells, respectively (Figure 3). These results suggest decreased PpIX synthesis rate or its faster degradation in resistant cells compared to sensitive cells. The analysis of heme metabolism enzymes showed changes in PPOX and FECH levels in tested resistant cell lines and no differences in the level of HO-1 (Figure 4). This confirms that decreased level of PS in resistant cells is a result of changes in heme metabolism. 

Decreased level of PPOX, the enzyme responsible for PpIX production, may lead to the reduction of PS synthesis rate in CAL-39 resistant cells. At the same time, the conversion of PpIX to heme could also be slowed down due to decreased FECH level. The decrease in PpIX synthesis and heme may also explain no increase in HO-1 expression in response to PDT. HO-1 is induced in response to increased free heme level in cells due to the necessity of its degradation [10,20]. HO-1 can also be induced in response to ROS and participate in the protection of cells against oxidative stress [20,21]. However, the observed increased level of HO-1 in sensitive CAL-39 cells suggests that higher expression of this enzyme is not sufficient to develop resistance to PDT. Moreover, although HO-1 plays a cytoprotective role in cancer cells, very high activity of this enzyme may also lead to ferroptosis as a result of Fe^2+^ overload and massive ROS production. Activity of HO-1 increases the level of cellular iron and promotes the production of ferritin. However, under oxidative stress, the ability of ferritin to bind to Fe^2+^ is impaired, potentially leading to uncontrolled release of Fe^2+^ into the cytosol [21]. 

On the other hand, in A-431 resistant cells, increased levels of PPOX and FECH enzymes were observed. Higher PPOX level can lead to an increase in PpIX synthesis rate, and higher amount of FECH is responsible for its immediate conversion to heme. Increased PpIX production coupled with its further conversion to heme may lead to reduced PS level in resistant cells. On the other hand, lack of changes in HO-1 level suggests that the rate of heme degradation has not increased. High concentration of free heme is toxic. However, it can be incorporated into hemeproteins, involved among others in mitochondrial respiration (cytochromes), cellular antioxidant defense (catalases and peroxidases) [20,22], and signal transduction processes (nitric oxide synthases, e.g., iNOS) [22]. It is also possible that resistant cells degrade excess heme through increased activity of heme oxygenase-2 (HO-2), as heme may regulate the activity of this protein. Additionally, the amount of this enzyme in cells and its modifications may influence the cellular level of heme oxygenase activity [23]. 

Apart from the accumulation capacity, the intracellular localization of PS (Figure S2, Supplementary Material) plays an important role in resistance development [10]. PpIX is a hydrophobic photosensitizer that accumulates in lipid structures of cancer cells [24]. However, PDT may also lead to protein and DNA damage [25]. The repair of damaged structures and molecules after PDT may be important for the development of resistance. In the CAL-39 cell line, the acquisition of resistance was accompanied by, among others, increased levels of proteins involved in DNA and membrane repair. These cells also showed increased levels of cytoplasmic and nuclear chaperones and proteins involved in proteasomal degradation (Figure 5b). Both the chaperones and proteins involved in proteasomal degradation may contribute to the reduction of PDT effects [10]. Molecular chaperones assist in the folding of unfolded and misfolded polypeptides. Several chaperones also function to reactivate aggregated proteins [26]. The cytoprotective role of the ubiquitin–proteasome system (UPS) results from degradation of oxidatively modified proteins [10]. 

Decreased levels of ER proteins involved in protein folding and increased levels of other molecular chaperones were also observed in the A-431 resistant cell line. An increased level of ST13, a co-chaperone of HSP70 family proteins, in both resistant cell lines may suggest that this protein, together with HSP70, may contribute to the reduction of the PDT effect. It was previously described that PDT induces a wide panel of different HSPs. HSP70, besides binding to damaged proteins and functioning as a chaperone, may have a cytoprotection role against PDT-induced apoptosis as it prevents the recruitment of procaspase-9 to the apoptosome complex [27].

Moreover, A-431 resistant cells showed decreased levels of pro-apoptotic proteins that can be activated, among others, by FAS death receptor [28,29]. Previous studies have shown that A-431 cell line increases the FAS level in response to PDT, which may lead to cell death in the extrinsic apoptotic pathway. Death receptor-induced apoptosis preferentially occurs when PS targets the cell membrane [27]. 

Although DNA photodamage was not directly linked with cell death caused by PDT, DNA oxidation may lead to mutations [25]. An increased amount of PpIX in the perinuclear area may cause higher accumulation of PS in the nucleus. This, in turn, may lead to increased DNA damage and major changes in cells subjected to PDT. Moreover, a high level of DNA damage may explain the increased levels of DNA repair proteins and increased activity of APE1 in resistant CAL-39 cells.

In addition, APE1 activity was even further increased after PDT (Figure 6). These results may confirm the importance of APE1 protein in acquiring resistance to PDT and in reducing the effects of therapy in CAL-39 resistant cells. However, APE1 activity was increased after PDT in both sensitive parental and resistant A-431 cell lines (Figure 6). The obtained results suggest that APE1 activity may be important not only for development of resistance in CAL-39 cells, but also for the elimination of DNA damage caused by PDT in both resistant cell lines. Moreover, inhibition of APE1 endonuclease activity efficiently sensitizes the resistant cells to PDT treatment. The use of an APE1 inhibitor with PDT decreased survival in both PDT resistant cell lines: CAL-39 and A-431 (Figure 7). This suggests a positive effect of combined PDT treatment with APE1 inhibitor against PDT resistance in VIN and vulvar cancer. 

Additionally, only one of three tested non-neoplastic cell lines showed decreased survival after PDT treatment combined with APE1 inhibitor, which may suggest a low toxicity of the combined therapy on non-neoplastic cell lines (Figure 8). The literature data also suggest the absence or low toxicity of small molecule inhibitors of the APE1 repair function in combination with temozolomide for normal cells, whereas they increase the sensitivity of cancer cells to therapy [30]. In this case, the decrease in survival after AR03 may be due to inhibition of cell division in non-cancer cells, rather than cell death. The effective sensitization of PDT-resistant cells with inhibitor of endonuclease activity of APE1, and likely low toxicity of such therapy for non-neoplastic cells, indicate a further research direction of combined treatment in vulvar neoplasms. 

However, the applied APE1 inhibitor may not only inhibit DNA repair in the BER pathway, but also may affect the regulation of gene expression. Inhibition of APE1 lyase function leads to changes in the transcription of genes activated by oxidative DNA damage (G-quadruplex structure formation) [31], or requiring repair before initiation of transcription (e.g., c-MYC-dependent genes) [16]. In addition, APE1, through its endonuclease domain, probably participates in RNA quality control, mRNA degradation [32,33,34], and miRNA processing [32,34]. Therefore, inhibition of the APE1 endonuclease may affect gene expression, including gene coding proteins involved in the heme metabolism pathway (Figure 9). These changes may also affect PDT sensitivity. 

The positive effect of an APE1 inhibitor on PDT in resistant A-431 cells may have been due to a decrease in HO-1 level. Taking into account that heme oxygenase activity depends on the level of both HO-isoforms in cells, a reduced level of HO-1 may decrease the rate of heme degradation and the cells’ protection ability against oxidative stress, even if HO-2 activity has been increased. In contrast, in resistant CAL-39 cells, the AR03 inhibitor may increase the effectiveness of PDT by increasing the level of FECH, which converts PpIX to heme. Due to its iron content, heme is not PS, but it can react with H_2_O_2_ and produce reactive oxygen intermediates whose nature depends on the degree of iron oxidation. The heme with Fe^2+^ catalyzes the formation of HO^•^, while the heme with Fe^3+^ generates porphyrin radical cation (PS^•+^) [35].

Taking into consideration that APE1, as a pro-survival factor, requires both redox and endonuclease activities [36], inhibition of redox function could also have a favorable effect on PDT sensitivity. APE1 through the redox domain can regulate the DNA binding capacity of various transcription factors, e.g., NF-κB, HIF-1α, AP-1, p53, [36,37,38], STAT-3 [38], NRF2, and HSF1 [37] to promote growth, migration, survival, inflammation, and angiogenesis [38]. This may lead to increased resistance to anti-cancer therapies, including PDT. Studies in A549 and HeLa tumor cell lines showed increased efficacy of PDT in combination with E3330, an inhibitor of APE1 redox function [37]. However, the potentially positive effect of redox inhibition on PDT in vulvar cancer cells requires careful investigation. 

In summary, this study shows that long-term use of photodynamic therapy may lead to the development of resistance in vulvar neoplastic cells. Reduction of the accumulation capacity of protoporphyrin IX is essential for acquiring resistance and may result from changes in the level of enzymes of the heme metabolic pathway. Additionally, changes in photosensitizer accumulation sites may influence the mechanisms leading to resistance in PDT-treated cells. Although the mechanisms leading to the development of resistance may vary even in the same type of cells, some features of PDT-resistant cells are common and have the potential to be used to sensitize them. Increased APE1 activity following therapy is not sufficient to develop resistance; however, along with other changes, it may lead to a weaker response to PDT. Increased activity of proteins involved in the DNA oxidative damage repair pathway is important to counteract the effects of PDT and may promote resistance to this therapy. Therefore, inhibition of APE1 activity may increase the efficacy of PDT. Resistant vulvar cancer cells can be effectively sensitized to PDT with APE1 endonuclease inhibitor. However, the APE1 inhibitor may improve the effects of therapy not only by reduction of DNA repair efficacy, but also by affecting gene expression, including genes coding enzymes of heme metabolism pathway. 

The obtained results are significant as they indicate a future research direction for PDT treatment combined with inhibition of DNA repair. This may contribute to invention of more effective treatment for VIN patients resistant to PDT.

## 4. Materials and Methods

### 4.1. Cell Line and Cell Culture

Two human vulvar squamous cancer cell lines were used as a VIN model. A-431 was purchased from ATCC and CAL-39 from DSMZ. Both cell lines had an elevated number of high affinity EGF receptors, showing an amplification of DNA sequences at 11q13 [39] where cyclin D1 resides [40]. Both cell lines had also mutated TP53 gene, and A-431 cell line showed additional mutations in CDKN2A and FBXW7 [41]. Cell lines sensitive and resistant to PDT A-431 were maintained in complete RPMI-1640 medium (Gibco by Thermo Fisher Scientific, Waltham, MA, USA) supplemented with 10% fetal bovine serum (FBS; Gibco) and 1% penicillin–streptomycin antibiotic solution (Pen-Strep; HyClone by Thermo Fisher Scientific, Waltham, MA, USA). Sensitive and resistant CAL-39 cells were cultured in Dulbecco’s modified Eagle’s medium (DMEM; HyClone) with 20% FBS, 2 mM L-glutamine (Gibco), 1 mM sodium pyruvate (Gibco), 0.5 nM hydrocortisone (Sigma-Aldrich, Saint Louis, MO, USA), 1 μg/100 mL EGF (Gibco), and 1% Pen-Strep.

For toxicity analysis, three non-cancer cell lines were used: (1) epithelial cell line MCF10A; (2) embryonic kidney cell line HEK293T; and (3) K21 cell line, which was immortalized from primary human fibroblasts with hTERT telomerase. HEK293T and K21 cells were cultured in DMEM with 10% FBS, 4 mM L-glutamine, and 1% Pen-Strep. MCF10A cells were maintained in 2/3 DMEM medium containing 10% donor horse serum (DHS) (Biowest, Nuaillé, France) and 1/3 MEGM medium (Lonza, Basel, Switzerland). 

All cell lines were cultured at 37 °C in a humidified incubator with 5% CO_2_ and atmospheric oxygen concentration.

### 4.2. Isolation of Cancer Cell Lines Resistant to PDT 

**Figure 10:**
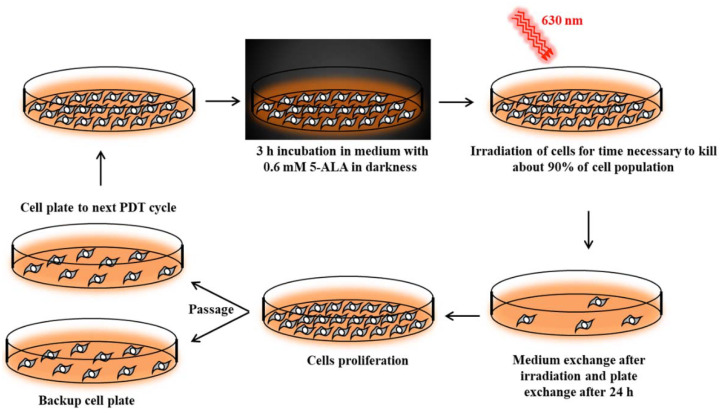
Isolation of PDT-resistant cells.

Cancer cell lines sensitive to PDT were subjected to repetitive cycles of PDT (Figure S1a,b, Supplementary Materials) according to Casas et al [42]. Cells were incubated for 3 h in serum-free medium with 0.6 mM ALA (Sigma-Aldrich). This incubation time was long enough to uptake 5-ALA and synthesize PpIX by cells [42]. Then, cells were exposed to red light (630 nm wavelength, 187 W/m^2^ power density) emitted by an LED lamp with a dose appropriate to kill about 90% of the cells (Figure 10). Surviving cells were cultured in growth medium until they reached 90% confluence and then were subjected to the next PDT treatment. This allowed for the isolation of PDT-resistant cells. The A-431 resistant cell line was isolated after 10 cycles of PDT, and the CAL-39 resistant cell line after 7 cycles.

### 4.3. PDT Treatment 

Cells were seeded in 96-well culture plates (A-431 cells at the density of 6 × 10^3^ cells per well and CAL-39 cell lines—8 × 10^3^ cells per well) 24 h before the experiment. Then, cells were incubated for 3 h with 0.6 mM 5-ALA in a serum-free medium and illuminated with increasing light doses: 16.8–101 J/cm^2^ for A-431 cell lines and 3.4–28.1 J/cm^2^ for CAL-39 cell lines. At the same time, untreated control cells were kept in the dark in serum-free medium. After irradiation (187 W/m^2^), the medium was replaced with a cultured one, and after the next 16 h, a cell viability assay was performed.

### 4.4. APE1 Inhibitor Treatment 

Inhibition of APE1 endonuclease activity was performed using AR03 (Axon Medchem, Groningen, The Netherlands), the specific inhibitor of APE1 activity. Cells were seeded in 96-well culture plates (A-431 cells at the density of 6 × 10^3^ cells per well; CAL-39, MCF10A, HEK293T, and K21 cell lines—8 × 10^3^ cells per well) 24 h before the experiment. Then, cells were incubated with AR03 for 19 h. AR03 concentrations used for A-431 resistant cells were 0–100 μM, for CAL-39 resistant cells—0–40 μM, and for non-cancer cells—0–20 μM. Afterwards, cell viability assay was performed.

### 4.5. Combined Treatment with PDT and APE1 Inhibitor

Cells were seeded in 96-well culture plates 24 h before the experiment (plated as before). Cells were incubated for 3 h with 5-ALA and AR03 (in concentrations described before) in serum-free medium. Then, cells were irradiated with increased light doses: for A-431 resistant cells—11.2–44.9 J/cm^2^; for CAL-39 resistant cells—3.4–16.8 J/cm^2^; for MCF10A cell line—2.5–7.6 J/cm^2^; HEK293T and K21 cell lines—0.7–2.1 J/cm^2^. Power density for resistant to PDT cancer cell lines was 187 W/m^2^, for MCF10A—84 W/m^2^, and for HEK293T and K21 cell lines—23 W/m^2^. Afterwards, the medium in all plates was replaced for 16 h with fresh culture medium supplemented with appropriate concentrations of AR03. When incubation time was over, cell viability assay was performed.

### 4.6. Cell Viability Assay 

Cell survival after treatment was measured with alamarBlue Cell Viability Reagent (Invitrogen Thermo Fisher Scientific). Medium of treated cells was exchanged to a fresh one supplemented with 10% alamarBlue. Cells were incubated 5 h at 37 °C in a cell culture incubator. Then, fluorescence intensity was measured using a DTX 880 Multimode Detector (Beckman-Coulter; Brea, CA, USA) at 540 nm excitation and 590 nm emission wavelengths. 

The fluorescence ratio of each tested group to untreated control was calculated and presented as a percentage value of the control. Cell viability assays were performed at least three times with independent cell cultures. 

### 4.7. Accumulation and Extraction of Protoporphirins

The day before ALA treatment, cells were seeded on 6 cm plates at a density of 8 × 10^5^ for A-431 cell lines or 4 × 10^5^ for CAL-39 cell lines. ALA-treatment was performed by incubation of cells (3–24 h) in 4 mL serum free-medium with 0.6 mM 5-ALA. Untreated cells were used as a control and to count the cell population. Measurement of porphyrin accumulation was performed twice by chemical extraction of intracellular porphyrins with 5% HCl. These conditions are optimal for total porphyrin extraction [42]. For measurement of porphyrin extraction, media were collected and acidified with an equal volume of 10% HCl. Then, cells were incubated twice for 30 min with 2 mL 5% HCl in 37 °C. Collected samples were subjected to fluorescence measurements using a Cary Eclipse fluorescence spectrophotometer (Agilent) at 406 nm excitation and 604 nm emission wavelengths, which corresponded to the highest emitted fluorescence of PpIX, uroporphyrin, and coproporphyrin solutions in 5% HCl [42]. Serial dilutions of PpIX (Frontier Scientific, Logan, UT, USA) in 5% HCl were used to plot a standard curve (Figure S3, Supplementary Materials). The amount of porphyrin in the cells and in the media was shown in ng/10^6^ cells. The experiment was performed in triplicate using independent cell cultures.

### 4.8. Proteome Comparison of Resistant to PDT Cell Lines and Parental Lines 

#### 4.8.1. Protein Extract Isolation

Cells were grown in 15 cm dishes to 80–90% confluency, washed 3 times with cold PBS, and centrifuged (5 min, 2000× *g* at 4 °C). A total of 9 M urea buffer (9 M urea, 0.15 M β-mercaptoethanol, 50 mM Tris-HCl; pH 7.4) was added to the cell pellet. Samples were vortexed, sonicated on ice, and incubated for 1 hour at −20 °C. Protein concentrations were measured by the Bradford method using protein assay reagent (Bio-Rad, Hercules, CA, USA). Protein extracts were isolated from three independent cell cultures. 

#### 4.8.2. Whole Cell Proteomics with LC-MS/MS

The 5 μL samples containing 17–29 μg of proteins were provided to the Laboratory of Mass Spectrometry (IBB PAS, Warsaw), where they were subjected to standard trypsin digestion, measured in an online LC-MSMS setup: NanoAcquity UPLC System (Waters, Milford, MA, USA) coupled to the Q Exactive Orbitrap Mass Spectrometer (Thermo Fisher Scientific, San Jose, CA, USA), operated in a data-dependent acquisition manner. Data were analyzed on the Mascot (MatrixScience, London, UK) and MaxQuant platforms (Jurgen Cox and Mathias Mann software [43]) using the reference Uniprot human proteome database. Statistical analysis was performed using the Scaffold 4Q + S (Proteome Software, Portland, OR, USA) platform. Proteins with *t*-test value ≤ 0.05 and fold change of resistance to sensitive cell ≥ 1.5 were taken for further analysis. A total of 85 of such proteins were detected in the A-431 cell line, and 390 in the CAL-39 cell line.

### 4.9. Immunofluorescence 

Cells were seeded on 24-well plates and cultured until 70% confluence was reached. Subsequently, cells were treated with (1) PDT, (2) APE1 inhibitor, or (3) PDT with APE1 inhibitor. For PDT treatment, cells were incubated 3 h with 0.6 mM 5-ALA and illuminated with a light dose of 3.9 J/cm^2^ for A-431 cell line and 1.1 J/cm^2^ for CAL-39 cell line. Afterwards, cells were incubated 6 h in culture media. For APE1 inhibitor treatment, cells were incubated for 9 h with 20 or 40 μM of AR03 for the A-431 cell line and 10 or 20 μM of AR03 for the CAL-39 cell line. For combined treatment of PDT with APE1, cells were firstly incubated 3 h with 5-ALA and AR03, illuminated, and then incubated for 6 h with APE1 inhibitor. Concentrations of 5-ALA and AR03 were used as described above. Moreover, light doses used in combined treatment were the same as in PDT treatment. Untreated cells were used as a control. In the next step, cells were washed 3 times with PBS, fixed 10 min at room temperature in PBS containing 3.7% paraformaldehyde, and permeabilized for 15 min with 0.2% Triton X-100 solution in PBS. Then, cells were washed 3 times with PBS and blocked for 1 h in SuperBlock (PBS) (Thermo Scientific) supplemented with 0.025% Triton. Cells, after washing 5 times with PBS containing 0.5% BSA, were incubated overnight with primary antibodies (Table 1). The next day, cells were washed 5 times with PBS containing 0.5% BSA and incubated for 1 h at room temperature in the dark with secondary antibodies (Table 1) diluted in PBS containing 0.5% BSA.

From that moment on, all activities were carried out with limited access to light. Samples were washed 5 times with PBS containing 0.5% BSA followed by 3 times with PBS. DAPI was dropped into each well and spread by tilting the plate in each direction followed by incubation for 5 min at room temperature. Cells were stored in PBS in a refrigerator until microscope observation. Six random pictures of a single well were taken using a Zeiss LSM 800 confocal microscope (20× objective). Images were analyzed with ImageJ software (National Institutes of Health, Bethesda, MD, USA), and CTCF was calculated. The experiment was carried out at least in triplicate using independent cell cultures. Results showing changes that occurred with the acquisition of resistance and in response to PDT in sensitive and resistant cells are presented as percentage values of sensitive cell fluorescence. Results of APE1 inhibitor treatment alone and in combination with PDT are presented as percentage values of untreated resistant cell fluorescence. 

### 4.10. APE1 Endonuclease Activity

#### 4.10.1. Cell Extract 

Cells were grown to 80–90% confluence in 15 cm dishes and subjected to PDT (3 h incubation with 0.6 mM 5-ALA and illumination with 16.8 J/cm^2^ light dose) or untreated. Then, cells were cultured in standard conditions for 3.5 h; washed 3 times with cold PBS; and centrifuged for 3 min, 4000× *g*, at 4 °C. Cell precipitates were suspended in buffer I (10 mM Tris-HCl (pH 7.8), 200 mM KCl) supplemented with 0.25 mM PMSF (Sigma-Aldrich) and 1× protease inhibitor cocktail (Complete, Roche). For each 10^6^ cells, 10 μL of buffer I was used. Afterward, an equal volume of buffer II (10 mM Tris-HCl (pH 7.8), 200 mM KCl, 2 mM EDTA, 2 mM DTT, 40% glycerol, 0.2% Nonidet P-40) with 0.25 mM PMSF and 1× protease inhibitor cocktail was added. Cell lysis was performed with shaking at 4 °C for 1.5 h. Then, samples were centrifuged (15 min, 16,000× *g* at 4 °C). Protein extracts were collected, aliquoted into 10 µL samples, and stored at −80 °C. Protein concentration was measured by Bradford method using protein assay reagent (Bio-Rad). Protein extracts were isolated from three independent cell cultures and analyzed for AP site incision activity. 

#### 4.10.2. DNA Substrate

The substrate for AP site incision was an oligodeoxynucleotide (ODN) with single lesion simulating the AP site—tetrahydrofuran (THF). This ODN (CTGCAGCTGATGCGC (THF) GTACGGATCCCCGGGTAC) was synthesized and PAGE purified by Midland Certified Reagent Company (Midland, TX, USA) and was labeled at the 5′-end with ^32^P. The labeling reaction was performed in 20 µL of a solution containing 1× reaction buffer (USB Corporation, Cleveland, OH, USA), 50 pmol ODN with THF, 20 µCi (γ32P) ATP (Hartmann Analytic, Braunschweig, Germany), 10 U OptiKinase (USB Corporation), and nuclease-free water for 1 h at 37 °C and completed by inactivation at 90 °C for 5 min. Complementary ODN (100 pmol) with G against THF was annealed to labeled oligonucleotide in 100 μL reaction with buffer containing 50 mM Tris-HCl (pH 7.5), 50 mM NaCl, and 1 mM DTT. The reaction was performed by heating for 5 min at 95 °C and cooling down at room temperature. Micro Bio-Spin P-30 columns (Bio-Rad) were used to purify the double-stranded substrate. Double-stranded oligodeoxynucleotides were stored at 20 °C.

#### 4.10.3. Measurements of AP Endonuclease Activity

Increasing concentration of cell extracts were used for measurement of AP site incision activity (0.5–5 μg for A-431 cell lines and 0.25–2.5 μg for CAL-39 cell lines). Cell extracts were prepared in 15 μL by dilution to appropriate concentration with dilution buffer (10 mM Tris-HCl (pH 7.8), 200 mM KCl, 1 mM DTT, 1 mM EDTA, 20% glycerol). The reaction was started by adding 5 μL of mix containing 0.125 pmol of double-stranded DNA substrate and was performed in 1x reaction buffer (20 mM HEPES (pH 7.8), 5 mM EDTA, 70 mM KCl, 2 mM MgCl_2_, 2 mM DTT), followed by incubation at 37 °C for 30 min. The reaction was terminated by adding SDS and proteinase K (Sigma-Aldrich) to final concentrations of 0.5% and 230 μg/mL, respectively, and incubation at 55 °C for 30 min. Then, formamide solution (92% formamide, 20 mM EDTA, 0.05% bromophenol blue, 0.05% xylene cyanol) was added in one-third of final volume of mixture, and samples were heated at 85 °C for 5 min. Afterwards, samples were cooled on ice. Reaction without cell extract was used as a negative control. Enzymatic reaction products were separated on 20% polyacrylamide gel containing 7 M urea, scanned with PhosphorImager and visualized with FujiFilm FLA7000 software (Fujifilm, Tokyo, Japan). The ratio of product to substrate was calculated with the Multi Gauge V3.0 software (Fujifilm). The product rate as a function of cellular protein extract was plotted. Enzymatic activity was calculated from the linear part of the curve and presented as fmoles of product per hour of reaction and per µg of protein extract (fmol/h/µg of proteins).

## Figures and Tables

**Table 1:** Antibodies used in the immunofluorescence experiment. All antibodies were purchased from Abcam (Cambridge, England).

Primary Antibodies	Product Number	Dilution	Secondary Antibody
Anti-PPOX antibody (EPR10400)	ab170412	1:500	Goat anti-rabbit IgG H&L (Alexa Fluor 594) (ab150080)
Anti-FECH antibody (EPR8312)	ab137042	1:500	
Anti-heme oxygenase 1 antibody (EP1391Y)	ab68477	1:500	

## Data Availability

Not applicable.

## Supplementary Materials

The following supporting information can be downloaded at https://www.mdpi.com/article/10.3390/ijms23084117/s1.

## References

[1] Hillemanns P., Untch M., Dannecker C., Baumgartner R., Stepp H., Diebold J., Weingandt H., Prove F., Korell M. (2000). Photodynamic Therapy of Vulvar Intraepithelial Neoplasia Using 5-Aminolevulinic Acid. Int. J. Cancer.

[2] Tosti G., Iacobone A., Preti E., Vaccari S., Barisani A., Pennacchioli E., Cantisani C. (2018). The Role of Photodynamic Therapy in the Treatment of Vulvar Intraepithelial Neoplasia. Biomedicines.

[3] van Straten D., Mashayekhi V., de Bruijn H., Oliveira S., Robinson D. (2017). Oncologic Photodynamic Therapy: Basic Principles, Current Clinical Status and Future Directions. Cancers.

[4] Mroz P., Yaroslavsky A., Kharkwal G.B., Hamblin M.R. (2011). Cell Death Pathways in Photodynamic Therapy of Cancer. Cancers.

[5] Romiszewska A., Bombalska A. (2018). The Use of 5-Aminolevulinic Acid and Its Derivatives in Photodynamic Therapy and Diagnosis. Biuletyn Wojskowej Akademii Technicznej.

[6] Sachar M., Anderson K.E., Ma X. (2016). Protoporphyrin IX: The Good, the Bad, and the Ugly. J. Pharmacol. Exp. Ther..

[7] Wachowska M., Muchowicz A., Firczuk M., Gabrysiak M., Winiarska M., Wańczyk M., Bojarczuk K., Golab J. (2011). Aminolevulinic Acid (ALA) as a Prodrug in Photodynamic Therapy of Cancer. Molecules.

[8] Mossakowska B., Fabisiewicz A., Siedlecki J. (2021). Terapia fotodynamiczna—Znaczenie w onkologii. Postepy Biochem..

[9] Lucena S., Salazar N., Gracia-Cazaña T., Zamarrón A., González S., Juarranz Á., Gilaberte Y. (2015). Combined Treatments with Photodynamic Therapy for Non-Melanoma Skin Cancer. Int. J. Mol. Sci..

[10] Casas A., Di Venosa G., Hasan T., Batlle A. (2011). Mechanisms of Resistance to Photodynamic Therapy. Curr. Med. Chem..

[11] Was H., Dulak J., Jozkowicz A. (2010). Heme Oxygenase-1 in Tumor Biology and Therapy. Curr. Drug Targets.

[12] Dorjsuren D., Kim D., Vyjayanti V.N., Maloney D.J., Jadhav A., Wilson D.M., Simeonov A. (2012). Diverse Small Molecule Inhibitors of Human Apurinic/Apyrimidinic Endonuclease APE1 Identified from a Screen of a Large Public Collection. PLoS ONE.

[13] Shahmoradi Ghahe S., Kosicki K., Wojewódzka M., Majchrzak B.A., Fogtman A., Iwanicka-Nowicka R., Ciuba A., Koblowska M., Kruszewski M., Tudek B. (2021). Increased DNA Repair Capacity Augments Resistance of Glioblastoma Cells to Photodynamic Therapy. DNA Repair.

[14] Yang Z.-Z., Li M.-X., Zhang Y.-S., Xiang D.-B., Dai N., Zeng L.-L., Li Z.-P., Wang G., Wang D. (2010). Knock down of the Dual Functional Protein Apurinic/Apyrimidinic Endonuclease 1 Enhances the Killing Effect of Hematoporphrphyrin Derivative-Mediated Photodynamic Therapy on Non-Small Cell Lung Cancer Cells in Vitro and in a Xenograft Model. Cancer Sci..

[15] Kelley M.R., Logsdon D., Fishel M.L. (2014). Targeting DNA Repair Pathways for Cancer Treatment: What’s New?. Future Oncol..

[16] Amente S., Lania L., Avvedimento E.V., Majello B. (2010). DNA Oxidation Drives Myc Mediated Transcription. Cell Cycle.

[17] Kwaśny M. (2018). Metoda fotodynamicznego leczenia w dermatologii. Forum Dermatol..

[18] Casas A., Perotti C., Di Venosa G., Batlle A., Rapozzi V., Jori G. (2015). Mechanisms of Resistance to Photodynamic Therapy: An Update. Resistance to Photodynamic Therapy in Cancer.

[19] Mansoori B., Mohammadi A., Davudian S., Shirjang S., Baradaran B. (2017). The Different Mechanisms of Cancer Drug Resistance: A Brief Review. Adv. Pharm. Bull..

[20] Kumar S., Bandyopadhyay U. (2005). Free Heme Toxicity and Its Detoxification Systems in Human. Toxicol. Lett..

[21] Chiang S.-K., Chen S.-E., Chang L.-C. (2018). A Dual Role of Heme Oxygenase-1 in Cancer Cells. Int. J. Mol. Sci..

[22] Ryter S.W., Tyrrell R.M. (2000). The heme synthesis and degradation pathways: Role in oxidant sensitivity heme oxygenase has both pro- and antioxidant properties. Free Radic. Biol. Med..

[23] Duvigneau J.C., Esterbauer H., Kozlov A.V. (2019). Role of Heme Oxygenase as a Modulator of Heme-Mediated Pathways. Antioxidants.

[24] Sułkowski L., Pawełczak B., Chudzik M., Maciążek-Jurczyk M. (2016). Characteristics of the Protoporphyrin IX Binding Sites on Human Serum Albumin Using Molecular Docking. Molecules.

[25] Bacellar I., Tsubone T., Pavani C., Baptista M. (2015). Photodynamic Efficiency: From Molecular Photochemistry to Cell Death. Int. J. Mol. Sci..

[26] Camberg J.L., Doyle S.M., Johnston D.M., Wickner S. (2013). Molecular Chaperones. Brenner’s Encyclopedia of Genetics.

[27] Nowis D., Makowski M., Stokłosa T., Legat M., Issat T., Gołab J. (2005). Direct Tumor Damage Mechanisms of Photodynamic Therapy. Acta Biochim. Pol..

[28] Jung Y.-S., Kim K.-S., Dong Kim K., Lim J.-S., Kim J.-W., Kim E. (2001). Apoptosis-Linked Gene 2 Binds to the Death Domain of Fas and Dissociates from Fas during Fas-Mediated Apoptosis in Jurkat Cells. Biochem. Biophys. Res. Commun..

[29] Kaufmann T., Strasser A., Jost P.J. (2012). Fas Death Receptor Signalling: Roles of Bid and XIAP. Cell Death Differ..

[30] Fishel M.L., Kelley M.R. (2007). The DNA Base Excision Repair Protein Ape1/Ref-1 as a Therapeutic and Chemopreventive Target. Mol. Asp. Med..

[31] Fedeles B.I. (2017). G-Quadruplex–Forming Promoter Sequences Enable Transcriptional Activation in Response to Oxidative Stress. Proc. Natl. Acad. Sci. USA.

[32] Antoniali G., Serra F., Lirussi L., Tanaka M., D’Ambrosio C., Zhang S., Radovic S., Dalla E., Ciani Y., Scaloni A. (2017). Mammalian APE1 Controls MiRNA Processing and Its Interactome Is Linked to Cancer RNA Metabolism. Nat. Commun..

[33] Li W.M., Barnes T., Lee C.H. (2010). Endoribonucleases—Enzymes Gaining Spotlight in MRNA Metabolism: Endoribonucleases in MRNA Metabolism. FEBS J..

[34] Tell G., Wilson D.M., Lee C.H. (2010). Intrusion of a DNA Repair Protein in the RNome World: Is This the Beginning of a New Era?. Mol. Cell. Biol..

[35] Vincent S.H. (1989). Oxidative Effects of Heme and Porphyrins on Proteins and Lipids. Semin. Hematol..

[36] Tell G., Fantini D., Quadrifoglio F. (2010). Understanding Different Functions of Mammalian AP Endonuclease (APE1) as a Promising Tool for Cancer Treatment. Cell. Mol. Life Sci..

[37] Franchi L.P., de Freitas Lima J.E.B., Piva H.L., Tedesco A.C. (2020). The Redox Function of Apurinic/Apyrimidinic Endonuclease 1 as Key Modulator in Photodynamic Therapy. J. Photochem. Photobiol. B Biol..

[38] Shah F., Logsdon D., Messmann R.A., Fehrenbacher J.C., Fishel M.L., Kelley M.R. (2017). Exploiting the Ref-1-APE1 Node in Cancer Signaling and Other Diseases: From Bench to Clinic. npj Precis. Oncol..

[39] Gioanni J., Grosgeorge J., Zanghellini E., Mazeau C., Gaudray P., Ettore F., Formento P., Demard F. (1993). Characterization of cal39, a new human cell-line derived from a vulvar squamous-cell carcinoma. Int. J. Oncol..

[40] Droll S., Bao X. (2021). Oh, the Mutations You’ll Acquire! A Systematic Overview of Cutaneous Squamous Cell Carcinoma. Cell. Physiol. Biochem..

[41] Zięba S., Kowalik A., Zalewski K., Rusetska N., Goryca K., Piaścik A., Misiek M., Bakuła-Zalewska E., Kopczyński J., Kowalski K. (2018). Somatic mutation profiling of vulvar cancer: Exploring therapeutic targets. Gynecol. Oncol..

[42] Casas A., Perotti C., Ortel B., Di Venosa G., Saccoliti M., Batlle A., Hasan T. (2006). Tumor Cell Lines Resistant to ALA-Mediated Photodynamic Therapy and Possible Tools to Target Surviving Cells. Int. J. Oncol..

[43] Cox J., Mann M. (2008). MaxQuant Enables High Peptide Identification Rates, Individualized p.p.b.-Range Mass Accuracies and Proteome-Wide Protein Quantification. Nat. Biotechnol..

