# Supplementary material for: Mechanisms of Resistance to Photodynamic Therapy (PDT) in Vulvar Cancer
Source: Int J Mol Sci. 2022 Apr 8;23(8):4117. doi: 10.3390/ijms23084117 (PMC9028356; doi:10.3390/ijms23084117)
Supplement: Supplementary file 1 [file ijms-23-04117-s001.zip › ijms-1640632-Supplementary Materials.pdf]

Mechanisms of resistance to photodynamic therapy (PDT) in vulvar cancer

Beata J. Mossakowska [1,] *, Somayeh Shahmoradi Ghahe [2], Dominik Cysewski [2], Anna Fabisiewicz [1], Barbara Tudek [2, 3,] ^ and Janusz A. Siedlecki [1]

[1] Department of Molecular and Translational Oncology, Maria Skłodowska-Curie National Research Institute of Oncology, Roentgena 5, 02-781 Warsaw, Poland; beata.mossakowska@pib-nio.pl; anna.fabisiewicz@pib-nio.pl; janusz.siedlecki@pib-nio.pl
[2] Institute of Biochemistry and Biophysics, Polish Academy of Sciences, Pawińskiego 5a, 02-106, Warsaw, Poland; s.shahmoradi@ibb.waw.pl; dominik.cysewski@ibb.waw.pl
[3] Faculty of Biology, Institute of Genetics and Biotechnology, University of Warsaw, Pawińskiego 5a, 02-106, Warsaw, Poland
* Correspondence: beata.mossakowska@pib-nio.pl
^ Deceased

SUPPLEMENTARY MATERIAL

Isolation of PDT resistant cancer cells

To induce PDT resistance in sensitive A-431 and CAL-39 vulvar cancer cell lines, the cells were subjected few times to PDT with increasing light dose. Induction was completed after 10th and 7th cycle of PDT in A431 and CAL-39 cells respectively (Figure S1a and S1b). Further attempts to increase resistance in CAL-39 cell line did not lead to any results in cells with unchanged morphology (after 6th cycle of PDT) and in cells with a changed shape (after 7th cycle of PDT). Resistance to PDT did not induce any morphological changes in A-431 cells (Figure S1c)

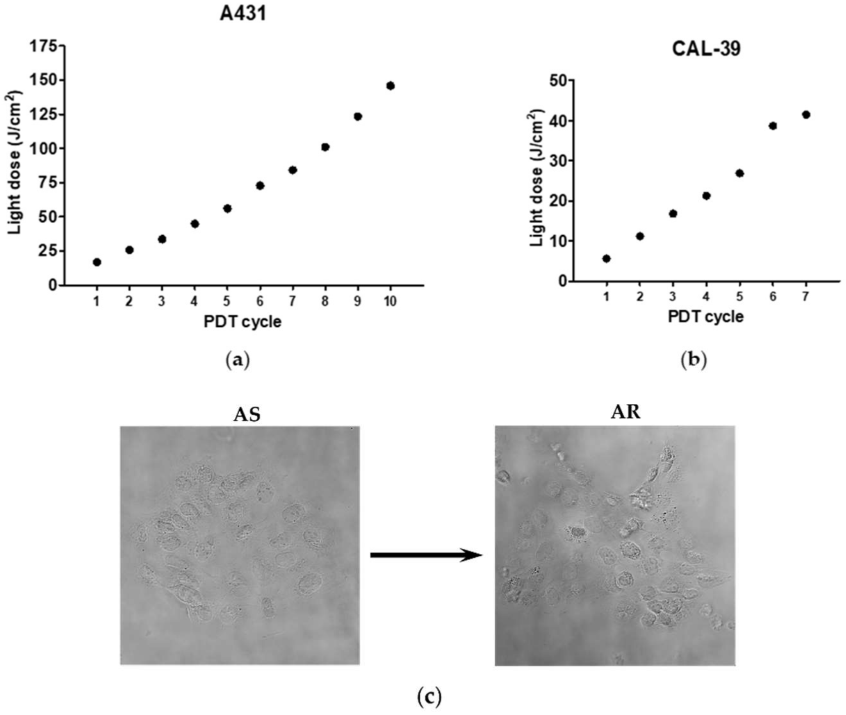

Figure S1. Induction of PDT resistance in sensitive cells. (**a**) Resistant cells from the A-431 cell line were isolated after 10 cycles with a light dose of 101 J/cm^2, which corresponds to more than 2 hours of irradiation; (**b**) Isolation of CAL-39 resistant cells was completed on cycle 7; with a light dose of 41.5 J/cm^2 that corresponds to 37 min of irradiation. (**c**) Shape of A-431 sensitive and resistant cells.

Porphirin visualisation

Decreased PDT efficacy may also result from changes in the localization of PS. PpIX is synthesized in mitochondria and can be accumulated in cell membranes. On the other hand, uroporphyrin and coproporphyrin, which are poor photosensitizers, as hydrophilic porphyrins may localize in cytoplasm. PpIX, uroporphyrin and coproporphyrin have similar excitation and emission wavelengths. Porphyrins visualization was performed after 24 h incubation of cell with ALA. Cells were also incubated with MitroTacker Green and Hoechst 33342 to stain mitochondria and nucleus, respectively (Figure S2).

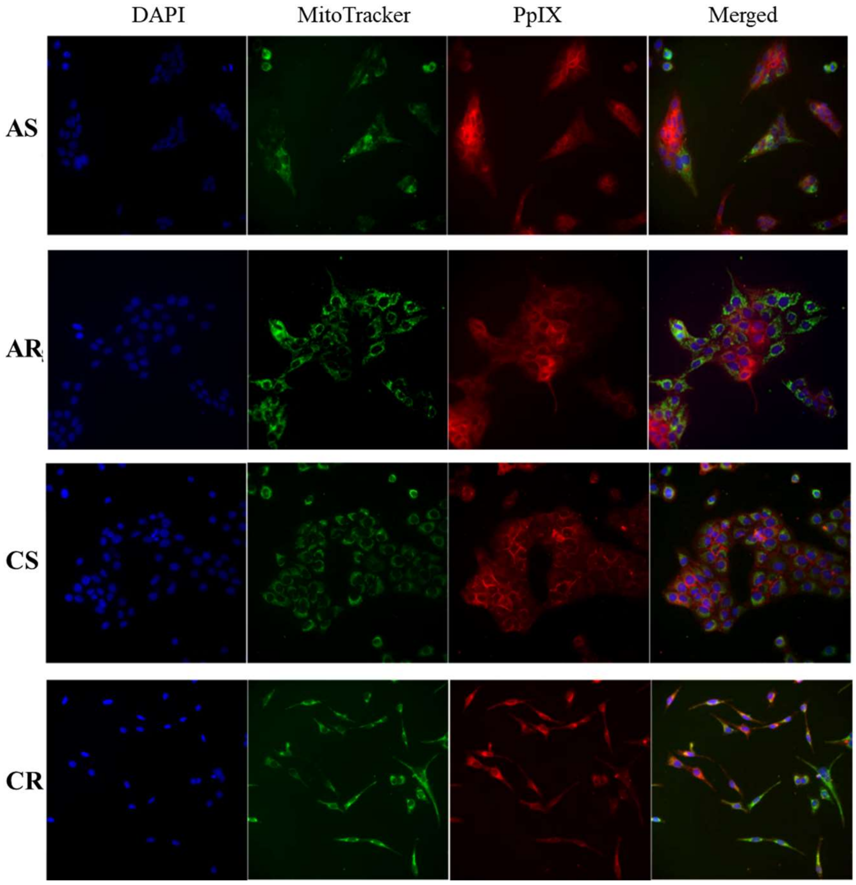

Figure S2. Porphyrin visualization. Cells were incubated 24 h in medium supplemented with 0.6 mM 5-ALA to visualized synthetized PpIX, then 30 min in serum free medium with 100 nM MitroTacker Green (Invitrogen) and 2.5 μg/ml Hoechst 33342 (Invitrogen) to stain mitochondria and nucleus respectively.

Standard curve for calculation of PpIX level

Serial dilutions of PpIX in 5% HCl or in 10% HCl mixed with equal volume of medium were used to plot a standard curve for calculation of protoporphyrin level in cells and media. Serial dilutions prepared before analysis of porphyrin accumulation and extraction in A-431 cells are shown on Figure S3a. PpIX concentration was calculated from the formula x = A/0.0552 for A431 cells and x = A/0.086 for RPMI medium. For analysis of porphyrins accumulation and extraction in CAL-39 cell line standard curves were prepared separately because of different time of analysis and another culture medium (Figure S3b). PpIX concentration was calculated as x = A/0.2565 for cells and x = A/0.2481 for DMEM medium.

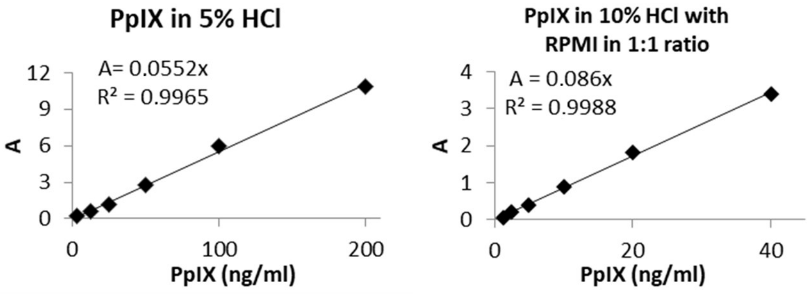

(**a**)

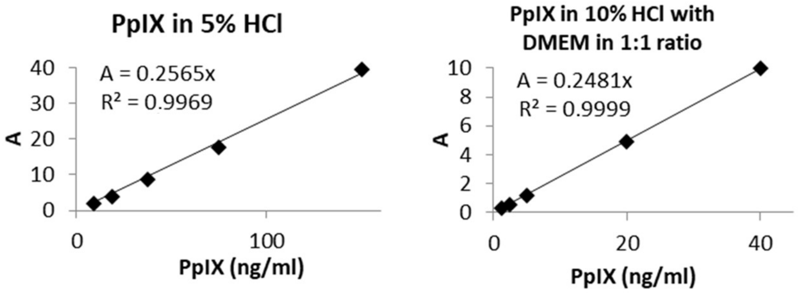

(**b**)

Figure S3. Standard curve for calculation of protoporphyrin level (**a**) in A431 cells and in RPMI medium; (**b**) in CAL-39 cells and in DMEM medium;

Proteome comparison of PDT resistant cell lines to their parental lines

Changes in the level of proteins involved in the repair after PDT and apoptosis

PDT may lead to damage of cell membranes, proteins and DNA. Repair of damaged structures and molecules after PDT may play a role in development the resistance. Changes in the level of proteins involved in these processes are presented in Table S1 for CAL-39 cell line and Table S2 for A-431 cell line.

As none of the cells were dying immediately after treatment, it can be assumed that these cells undergo programmed cell death after PDT. For this reason, changes in level of proteins involved in cell death pathways were also analyzed. Differences in amount of pro-apoptotic proteins are also presented in the Table S2 .

Table S1. Changes in the level of proteins involved in the repair after PDT and apoptosis in CAL-39 resistant cells

		Gen	T-Test	CR:CS
**Proteins involved in DNA repair**	**HR**	NUCKS1	0,017	Only in CR
	**HR**	BCCIP	0,00079	14
	**NHEJ**	HMGB2	0,0059	14
	**HR, NHEJ**	DEK	0,041	5,2
	**BER, NER, NHEJ**	NME1	0,023	5,1
	**BER, NER, MMR, NHEJ**	HMGB1	0,012	4,5
	**BER, NER, MMR, HR**	PCNA	0,026	4,3
	**BER, NER, HR, NHEJ**	PARP1	0,0043	4,1
	**BER, NIR**	APEX1	0,017	2,6
	**NHEJ**	XRCC6	0,045	2,4
	**HR, NHEJ**	TPT1	0,034	2,2
**Protein involved in membrane repair**		PDCD6IP	0,014	Only in CR
		ANXA5	0,00051	12
		CHMP4B	0,014	8,8
		MYOF	0,011	2

Cytoplasmic and nuclear molecular chaperones	Co-chaperones	CHORDC1	0,013	Only in CR
		CDC37	0,0038	130
		ST13	0,021	27
	HSP27	HSPB1	0,0049	7
	HSP70	HSPA2	0,017	5
	HSP90	HSP90AB2P	0,025	2,7
		HSP90AB1	0,011	2,2
		HSP90AA1	0,032	1,9
	TCTP	TPT1	0,034	2,2
		CCT5	0,021	0,3
	Peptidyl-Proline Isomerases (PPIases)	PIN1	0,00044	Only in CR
		FKBP3	0,036	Only in CR
		FKBP1A	0,0017	9,4

Table S2. Changes in the level of proteins involved in the repair after PDT and apoptosis in A-431 resistant cells

	Gen	T-Test	AR:AS
DNA repair	RUVBL2	0,038	4,1
Cytoplasmic/nuclear co-chaperones	ST13	0,00091	13
	STIP1	0,03	2,2
Apoptosis	BID	0,038	0,1
	PDCD6	0,049	0,3

Changes in the levels of proteins involved in synthesis, folding and degradation of proteins

An increased amount of cytoplasmic and nuclear molecular chaperones was linked with decreased level of proteins involved in protein processing in ER in both resistant cell lines. Both cell line showed also differences in level of proteins involved in translation and degradation of proteins. (Table S3 and S4).

In addition, CAL-39 resistant cells decreased level of cathepsin D (CTSD) and increased amount of cystatin B (CSTB) which may protect this cells from lysosomal cell death (Table S3). Cathepsin D is a lysosomal aspartyl protease that is involved in the proteolysis of intracellular and extracellular proteins and may be involved in LCD. Cystatin B, on the other hand, is an intracellular thiol protease inhibitor that is capable of inhibiting, among others, L, H, B cysteine cathepsins and may participate in the protection of cells against proteases leaking from the lysosomes.

Table S3. Changes in the level of proteins involved in protein synthesis, processing and degradation in CAL-39 resistant cells

			Gen	T-Test	CR:CS
Protein synthesis in cytoplasm	aa--tRNA ligases		GARS	0,0076	Only in CR
			VARS	0,019	93
			AARS	0,028	43
			NARS	0,019	33
			MARS	0,013	19
			QARS	0,0032	7,4
			DARS	0,023	6,4
			WARS	0,047	4
	Ribosome	40S	RPS29	0,043	21
			RPS15A	0,016	6,1
			RPS15	0,023	1,6
		60S	RPL23	0,047	5,6
			RPL35	0,035	3,1
			RPL7	0,014	2,3
			RPL3	0,0069	2
			RPL6	0,04	1,8
			RPL10	0,041	1,8
			RPL18	0,043	1,8
			RPL13	0,029	1,7

	**Initiation**	EIF4H	0,0073	Only in CR
		EIF5B	0,019	Only in CR
		EIF3I	0,0013	85
		EIF3H	0,00096	24
		EIF5A	0,00068	14
		EIF3A	0,0023	4,9
		EIF3CL	0,023	4,4
		EIF2S3	0,036	4,4
		EIF4G1	0,026	2,9
		EIF2S1	0,027	2,2
		EIF4A1	0,029	2
	**Elongation**	EEF2	0,00045	8,9
		EEF1G	0,012	4,6
		EEF1A1	0,0025	2,7
	**Termination**	ETF1	0,00091	Only in CR
		GSPT1	0,03	Only in CR
		GSPT2	0,022	33
**Protein processing in ER**	**Translocon**	SEC61B	0,0036	0,3
	**Ribosome anchor**	CKAP4	0,042	0,4
		RPN1	0,0088	0,3
	**Protein folding in ER**	CALR	0,016	1,9
		PDIA3	0,011	0,6
		HSPA5	0,0016	0,5
		HSP90B1	0,017	0,5
		CANX	0,029	0,5
		PDIA6	0,012	0,4
		GANAB	0,0016	Only in CS
	**ERAD**	NSFL1C	0,003	Only in CR
		CCDC47	0,025	0,2
		DERL1	0,0089	Only in CS
**Proteasomal degradation**	**Protein ubiquitination**	TRIM25	0,00041	Only in CR
		UBA1	0,00025	170
		CACYBP	0,01	27
		UBE2L3	0,0032	26
		CAND1	0,0077	7,3
	**Protein deubiquitination**	USP5	0,0021	Only in CR
		USP14	0,025	Only in CR
	**Proteasome assembly**	ECPAS	0,00012	Only in CR
	**Proteasome**	PSMB7	< 0,00010	Only in CR
		PSME3	0,0039	Only in CR
		PSMD3	0,0024	Only in CR
		PSMD2	0,0016	14
		PSMC2	0,0046	6,3
		PSMC6	0,036	5,2
		PSMA6	0,015	2,2
**Proteases**		ERAP1	0,0058	0,1
		HM13	0,0083	0,02
		CTSD	< 0,00010	0,02
**Inhibitor protease**		CSTB	0,013	Only in CR
**Protein modification**		PCMT1	0,0031	9,5
		SUMO2	0,021	1,5
**Mitochondrial protein synthesis**	**Ribosome 39S**	MRPL41	< 0,00010	Only in CS
		MRPL38	0,0014	Only in CS
		MRPL9	0,012	Only in CS
		MRPL47	0,023	Only in CS
	**Tranlation**	TSFM	0,0026	Only in CS

Mitochoondrial HSP	HSPD1	0,012	0,5
	HSPE1	0,027	0,4
	TRAP1	0,039	0,1

Table S4. Changes in the level of proteins involved in protein metabolism in A-431 resistant cells

		Gen	T-Test	AR:AS
Protein synthesis	aa--tRNA ligases	AARS	< 0,00010	Only in AS
	Initiation	EIF5B	0,028	2,2
		EIF3M	0,044	0,3
	Ribosome 39S	MRPL9	0,017	0,2
	Regulation	PUS7	0,043	Only in AS
Protein processing in ER		PDIA6	0,028	0,4
		MESD	0,003	Only in AS
		DNAJA2	0,031	Only in AS
Protein degradation	Regulation	UBQLN2	0,04	Only in AR
	Proteases	CTSC	0,037	6,2
		HM13	0,035	0,2
	Ubiquitination	UBE2L3	0,0062	0,4
	Proteasome	PSMA1	0,019	0,1
Calpain inhibitor		CAST	0,039	Only in AS

Changes in the level of proteins involved in calcium ion homeostasis

Second function of ER, apart from folding, modifying and sorting of newly synthesized proteins is Ca2+ storage and signaling. Most of ER proteins that are involved in protein processing (folding, ER-associated degradation - ERAD) and in Ca^{2+} homeostasis were decreased in CAL-39 resistant cells (Table S4 and S5). ER damage following PDT can result in dramatic changes in ER homeostasis, which can lead to mitochondrial cell death. ER localizing PS leads to release cellular deposits of calcium and extensive photodamage to the sarco/endoplasmic reticulum Ca2+ -ATPase-2 (SERCA2) pump which regulate Ca2+ influx to ER. SERCA2 (ATP2A2) and ASPH, which regulates ER Ca2+ efflux were decreased in CAL-39 resistant cells. Moreover CCDC47, ER protein involved in regulation of calcium ion homeostasis, was decreased and proteins involved in calcium ion homeostasis that localize in other cellular compartments were increased. Increased level of some calcium buffering proteins in various cellular compartments can protect cells from Ca^{2+}-induced cell death as intracellular Ca^{2+} overload may lead to initiation of mitochondrial apoptosis. Also ER Ca^{2+} depletion and incapability of the cells to refill ER Ca^{2+} pools may lead to initiation of intrinsic pathway of apoptosis, therefore decreased level of Ca^{2+} ER transport proteins may potentially affect the ability to initiate apoptosis.

Table S5. Changes in the level of proteins involved in protein synthesis, processing and degradation in CAL-39 resistant cells

	Localization	Gen	T-Test	CR:CS
Calcium ion homeostasis	Nucleus, ER, Golgi, Cytoplasm	NUCB2	0,04	9,3
	Mitochondrion	SLC25A24	0,0051	3,3
	Cytoplasm	TPT1	0,034	2,2
	ER, cytosol, cell surface	CALR	0,016	1,9
	ER	CCDC47	0,025	0,2
	ER	ASPH	0,027	0,03
	ER	ATP2A2	0,011	0,02

References

1. Kegg mapper https://www.genome.jp/kegg/mapper/search.html
2. Uniprot https://www.uniprot.org/
3. Genecard https://www.genecards.org/
4. Brandsma, I.; Gent, D.C. Pathway Choice in DNA Double Strand Break Repair: Observations of a Balancing Act. *Genome Integr* **2012**, *3*, 1–10, doi:10.1186/2041-9414-3-9.
5. Buytaert, E.; Dewaele, M.; Agostinis, P. Molecular Effectors of Multiple Cell Death Pathways Initiated by Photodynamic Therapy. *Biochimica et Biophysica Acta (BBA) - Reviews on Cancer* **2007**, *1776*, 86–107, doi:10.1016/j.bbcan.2007.07.001.
6. Camberg, J.L.; Doyle, S.M.; Johnston, D.M.; Wickner, S. Molecular Chaperones. In *Brenner's Encyclopedia of Genetics*; Elsevier, 2013; pp. 456–460 ISBN 978-0-08-096156-9.

7. Huang, P.; Cai, Y.; Zhao, B.; Cui, L. Roles of NUCKS1 in Diseases: Susceptibility, Potential Biomarker, and Regulatory Mechanisms. *BioMed Research International* **2018**, *2018*, 1–7, doi:10.1155/2018/7969068.
8. Kang, R.; Zhang, Q.; Zeh, H.J.; Lotze, M.T.; Tang, D. HMGB1 in Cancer: Good, Bad, or Both? *Clinical Cancer Research* **2013**, *19*, 4046–4057, doi:10.1158/1078-0432.CCR-13-0495.
9. Li, Y.; Sun, H.; Zhang, C.; Liu, J.; Zhang, H.; Fan, F.; Everley, R.A.; Ning, X.; Sun, Y.; Hu, J.; et al. Identification of Translationally Controlled Tumor Protein in Promotion of DNA Homologous Recombination Repair in Cancer Cells by Affinity Proteomics. *Oncogene* **2017**, *36*, 6839–6849, doi:10.1038/onc.2017.289.
10. Lu, H.; Yue, J.; Meng, X.; Nickoloff, J.A.; Shen, Z. BCCIP Regulates Homologous Recombination by Distinct Domains and Suppresses Spontaneous DNA Damage. *Nucleic Acids Research* **2007**, *35*, 7160–7170, doi:10.1093/nar/gkm732.
11. Mroz, P.; Yaroslavsky, A.; Kharkwal, G.B.; Hamblin, M.R. Cell Death Pathways in Photodynamic Therapy of Cancer. *Cancers* **2011**, *3*, 2516–2539, doi:10.3390/cancers3022516.
12. Puts, G.S.; Leonard, M.K.; Pamidimukkala, N.V.; Snyder, D.E.; Kaetzel, D.M. Nuclear Functions of NME Proteins. *Lab Invest* **2018**, *98*, 211–218, doi:10.1038/labinvest.2017.109.
13. Radić, M.; Šoštar, M.; Weber, I.; Ćetković, H.; Slade, N.; Herak Bosnar, M. The Subcellular Localization and Oligomerization Preferences of NME1/NME2 upon Radiation-Induced DNA Damage. *IJMS* **2020**, *21*, 1–19, doi:10.3390/ijms21072363.
14. Rajendra, E.; Garaycoechea, J.I.; Patel, K.J.; Passmore, L.A. Abundance of the Fanconi Anaemia Core Complex Is Regulated by the RuvBL1 and RuvBL2 AAA+ ATPases. *Nucleic Acids Research* **2014**, *42*, 13736–13748, doi:10.1093/nar/gku1230.
15. Ray Chaudhuri, A.; Nussenzweig, A. The Multifaceted Roles of PARP1 in DNA Repair and Chromatin Remodelling. *Nat Rev Mol Cell Biol* **2017**, *18*, 610–621, doi:10.1038/nrm.2017.53.
16. Raymond, A.-A.; Benhamouche, S.; Neaud, V.; Di Martino, J.; Javary, J.; Rosenbaum, J. Reptin Regulates DNA Double Strand Breaks Repair in Human Hepatocellular Carcinoma. *PLoS ONE* **2015**, *10*, 1–15, doi:10.1371/journal.pone.0123333.
17. Reeves, R.; Adair, J.E. Role of High Mobility Group (HMG) Chromatin Proteins in DNA Repair. *DNA Repair* **2005**, *4*, 926–938, doi:10.1016/j.dnarep.2005.04.010.
18. Shin, Y.-J.; Kim, M.-S.; Kim, M.-S.; Lee, J.; Kang, M.; Jeong, J.-H. High-Mobility Group Box 2 (HMGB2) Modulates Radioresponse and Is Downregulated by P53 in Colorectal Cancer Cell. *Cancer Biology & Therapy* **2013**, *14*, 213–221, doi:10.4161/cbt.23292.
19. Smith, E.A.; Gole, B.; Willis, N.A.; Soria, R.; Starnes, L.M.; Krumpelbeck, E.F.; Jegga, A.G.; Ali, A.M.; Guo, H.; Meetei, A.R.; et al. DEK Is Required for Homologous Recombination Repair of DNA Breaks. *Sci Rep* **2017**, *7*, 1–12, doi:10.1038/srep44662.
20. Sønder, S.L.; Boye, T.L.; Tölle, R.; Dengjel, J.; Maeda, K.; Jäättelä, M.; Simonsen, A.C.; Jaiswal, J.K.; Nylandsted, J. Annexin A7 Is Required for ESCRT III-Mediated Plasma Membrane Repair. *Sci Rep* **2019**, *9*, 1–12, doi:10.1038/s41598-019-43143-4.
21. Thapar, R. Roles of Prolyl Isomerases in RNA-Mediated Gene Expression. *Biomolecules* **2015**, *5*, 974–999, doi:10.3390/biom5020974.
22. Turtoi, A.; Blomme, A.; Bellahcene, A.; Gilles, C.; Hennequiere, V.; Peixoto, P.; Bianchi, E.; Noel, A.; De Pauw, E.; Lifrange, E.; et al. Myoferlin Is a Key Regulator of EGFR Activity in Breast Cancer. *Cancer Research* **2013**, *73*, 5438–5448, doi:10.1158/0008-5472.CAN-13-1142.
23. Vorster, L.; Abrahamse, H. *DNA Repair: New Research: Chapter 7 DNA Damage and Repair during Photodynamic Theraphy*; Kimura, S., Shimizu, S., Eds.; DNA and RNA : properties and modifications, functions and interactions, recombination and applications; Nova Science Publishers: New York, 2012; ISBN 978-1-62100-756-2.
24. Yadav, A.; Kumar, B.; Lang, J.C.; Teknos, T.N.; Kumar, P. A Muscle-Specific Protein 'Myoferlin' Modulates IL-6/STAT3 Signaling by Chaperoning Activated STAT3 to Nucleus. *Oncogene* **2017**, *36*, 6374–6382, doi:10.1038/onc.2017.245.
25. Yang, Z.-Z.; Li, M.-X.; Zhang, Y.-S.; Xiang, D.-B.; Dai, N.; Zeng, L.-L.; Li, Z.-P.; Wang, G.; Wang, D. Knock down of the Dual Functional Protein Apurinic/Apyrimidinic Endonuclease 1 Enhances the Killing Effect of Hematoporphrphyrin Derivative-Mediated Photodynamic Therapy on Non-Small Cell Lung Cancer Cells in Vitro and in a Xenograft Model. *Cancer Science* **2010**, *101*, 180–187, doi:10.1111/j.1349-7006.2009.01366.x.
26. Zhang, J.; de Toledo, S.M.; Pandey, B.N.; Guo, G.; Pain, D.; Li, H.; Azzam, E.I. Role of the Translationally Controlled Tumor Protein in DNA Damage Sensing and Repair. *Proceedings of the National Academy of Sciences* **2012**, *109*, E926–E933, doi:10.1073/pnas.1106300109.
